# A Preliminary Study of Copy Number Variation in Tibetans

**DOI:** 10.1371/journal.pone.0041768

**Published:** 2012-07-23

**Authors:** Yong-Biao Zhang, Xin Li, Feng Zhang, Duen-Mei Wang, Jun Yu

**Affiliations:** CAS Key Laboratory of Genome Sciences and Information, Beijing Institute of Genomics, Chinese Academy of Sciences, Beijing, People’s Republic of China; Seoul National University College of Medicine, Republic of Korea

## Abstract

Genetic features of Tibetans have been broadly investigated, but the properties of copy number variation (CNV) have not been well examined. To get a preliminary view of CNV in Tibetans, we scanned 29 Tibetan genomes with the Illumina Human-1 M high-resolution genotyping microarray and identified 139 putative copy number variable regions (CNVRs), consisting of 70 deletions, 61 duplications, and 8 multi-allelic loci. Thirty-four of the 139 CNVRs showed differential allele frequencies versus other East-Asian populations, with *P* values <0.0001. These results indicated a distinct pattern of CNVR allele frequency distribution in Tibetans. The Tibetan CNVRs are enriched for genes in the disease class of human reproduction (such as genes from the *DAZ*, *BPY2*, *CDY*, and *HLA-DQ* and *-DR* gene clusters) and biological process categories of “response to DNA damage stimulus” and “DNA repair” (such as *RAD51*, *RAD52*, and *MRE11A*). These genes are related to the adaptive traits of high infant birth weight and darker skin tone of Tibetans, and may be attributed to recent local adaptation. Our results provide a different view of genetic diversity in Tibetans and new insights into their high-altitude adaptation.

## Introduction

Environmental hypoxia, high ultraviolet (UV) radiation, low temperatures, and low precipitation are the harsh nature of the Tibetan Plateau. Modern Tibetans have adapted to live in these rigorous conditions, as evidenced by their efficient oxygen utilization, absence of chronic mountain sickness, and high infant survival rate [1]. Although it is known that Tibetans are a branch of East-Asian (EA) populations [2], [3], [4], long-term endurance in the harsh environment has facilitated the divergence of Tibetans from other East-Asian populations with a distinct set of genetic characteristics [5].

Studies on Tibetans have mostly focused on disease susceptibility [6], [7], migration history [2], [5], [8], and high-altitude adaptation [5], [9]. Great success was achieved with the use of genetic markers from SNP, STR, and haplotypes of mitochondria, as well as Y chromosomes. However, very few CNV studies were reported of Tibetans so far. DNA copy number variation is an important type of genomic variation that can influence phenotype through gene dosage effect and/or alteration of the genomic architecture [10], and is a good candidate for natural selection [11]. Copy number variable region (CNVR) usually contains higher nucleotide count per genome than that of SNP [12], and may contain more than one gene and/or their regulatory regions [10]. CNVs have been shown to account for nearly 18% of variation in gene expression [13]. In humans, breakpoints of a CNV preferentially possess sequence motifs related to chromosomal rearrangement or genome instability [14]. These suggest that the impacts of CNVs on genomes may easily turn into phenotypic alterations.

Recently, more CNVs have been implicated in pathogenesis, from rare genetic disorders to a wide-range of common diseases [15]. Most CNV-related diseases are systemic autoimmune diseases, mood and psychotic disorders, and AIDS [16], [17], [18], [19]. In addition to the pathogenic CNVs, extensive amounts of common CNVs have been identified from healthy individuals. Discovery studies of CNVs with diverse populations found significant differences in the frequencies of CNVs among distinct ethnic groups [12], [20]. With genotyping microarrays, the number of CNVs identified per individual genome ranged from 3.5 in African Americans [21] to 40.3 in Koreans [22]. Despite advances in discovery and functional studies, the exhaustion of the non-pathogenic CNV pool with larger sample sizes and more ethnic groups will portray a better genomic landscape in humans.

To characterize the allele frequency distribution of CNVs in Tibetans and to explore the role of CNVs in high-altitude adaptation, we scanned 29 Tibetan genomes, using high-resolution genotyping microarrays. This preliminary study will further improve our understanding of the genetic diversity of Tibetans and will benefit research on high-altitude adaptation and disease susceptibility of Tibetans.

## Results

### The General Characteristics of CNV and CNVR

**Figure 1:**
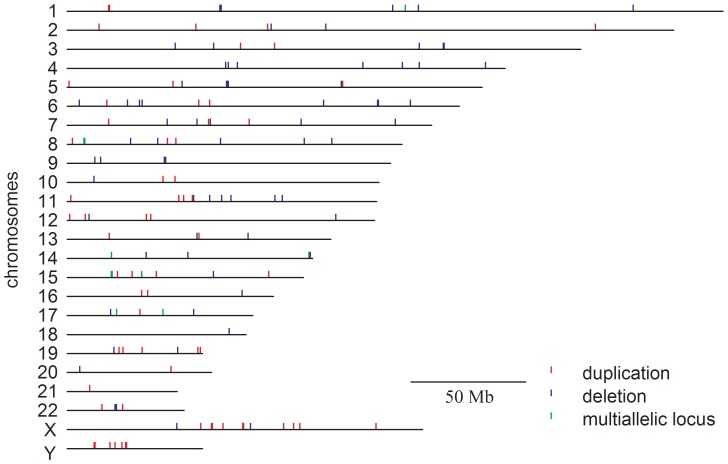
Structural variation map of Tibetan samples. The schematic summarizes the distribution of duplications, deletions and multi-allelic loci on each human chromosome.

With high-resolution genotyping microarrays, we identified 562 candidate CNVs from 29 Tibetans, with an average of 19.38 CNVs per genome and a corresponding average length of 6.07 Mb per genome. The mean size of our CNVs was 313 kb, ranging from 75.5 kb to 4.2 Mb. These 562 CNVs corresponded to 139 CNVRs (70 deletions, 61 duplications, and 8 multi-allelic loci), covering a total of 1.67 percent of the human genome (Figure 1, Table S1). Of the 139 CNVRs, 123 (88%) were found to be overlapped with CNVRs from the Database of Genomic Variants (DGV, latest updated: Nov 02, 2010) and were thus considered known CNVRs. About half of our known CNVRs (62 out of the 123 CNVRs) were common, with allele frequency greater than 5%. The remaining 16 CNVRs (12%; 12 deletions and 4 duplications) were novel, all with allele frequency less than 5% and 14 of them were validated by real-time quantitative PCR (Table S2). The 139 CNVRs encoded 344 genes as annotated by RefSeq (release 50), of which 184 genes had an Entrez gene summary. About 70% of our CNVRs overlapped with RefSeq genes, with no strong bias of harboring RefSeq genes between the deletion and duplication regions (78% and 57%, respectively).

### Compare with East-Asian Populations

To better understand the properties of CNVs found in Tibetans, we compared some relevant statistics of our results with those of East-Asian (EA) populations and HapMap Phase II Samples (Table 1) [20], [22], [23], [24], [25]. The analyzed EA populations were composed of 300 Han Taiwanese [25], 692 Han Chinese [20], 800 Han Taiwanese [23], and 3578 Koreans [22]. With the Human-1 M chip, we found that in Tibetans, the average sizes of CNVs (313 kb) and CNVRs (370.6 kb), as well as the average CNV coverage per genome (6.07 Mb), are larger than those in other EA populations (Table 1). Our results may be influenced by our detection platform, which is sensitive to the larger-sized CNVs [26]. Comparing the shared CNVRs between and among populations, we noticed that Tibetans share fewer CNVRs with Han Chinese than with other populations. In total, 74 shared CNVRs were found between Tibetan and other EA populations. Of the shared CNVRs, 25 showed significant differences in allele frequency (*P* values <0.0001; Table S1), and an additional 9 were exclusively present in Tibetans, all with high allele frequency (>10%). Combining these two observations, we extracted 34 CNVRs as a putative distinct set, of which allele frequency distributions were significantly deviated from other EA populations. This set of CNVRs may offer information for our subsequent analyses on the high-altitude adaptation of Tibetans.

**Table 1:** Characteristic summary of CNVRs found in Tibetan and other populations.

Population	Tibetan	Han Taiwanese [25]	Han Chinese [20]	Han Taiwanese [23]	Korean [22]	CHB+JPT [24]	CEU [24]	YRI [24]
Sample size	29	300	692	813	3578	90	90	90
Type of chips	Illu. 1 M	Affy. 100 k	Affy. 500 k	Illu. 550 k	Affy. 5.0	Affy. 6.0	Affy. 6.0	Affy. 6.0
Number of CNVRs	139	230	725	365	4003	646	657	916
Average size of CNV (kb)	313	186	299	107	18.9	ND	ND	ND
Average number of CNVsper individual genome	19.38	5.02	10	4.6	40.3	ND	ND	ND
Average CNV coverage per genome (Mb)	6.07	0.93	3.56	0.497	ND	ND	ND	ND
Average size of CNVR (kb)	370.6	322	356	235.1	60.4	35.5	35.12	25.5
Number of overlappedCNVRs with Tibetan	139	9	25	11	33	45	47	53
Number of reportedCNVRs in DGV	121	133	529	298	1926	646	657	916

Abbreviation: Illu., Illumina; Affy., Affymetrix; ND, No data; kb, kilo base; Mb, mega base; CNV, copy number variation; CNVR, copy number variation region; CHB, Han Chinese; JPT, Japanese; CEU, descendant of European; YRI, Yoruban.

To understand the Tibetan-specific biological effects from our CNVs, we focused on the aforementioned 34 CNVRs, including 18 duplications, 13 deletions, and 3 multi-allelic loci. Among the 34 CNVRs, 14 CNVRs overlapped with Entrez genes and are listed in Table 2. Each of the 14 CNVRs was larger than 100 kb, and over half of them (57%) were in the form of deletion. In addition, autosomal chromosomes had mostly deletion type of CNVRs, whereas sex chromosomes had mostly duplication. The allele frequency discrepancy of variants among populations can be affected by factors, such as selection, genetic drift, and gene flow. Still, local adaptation is a major selection force to shape the discrepancy among geographically close populations [27], [28]. Therefore, these 14 CNVRs that showed significant differences in allele frequency between Tibetan and other EA populations were potentially due to recent adaptation. Furthermore, we found 4 CNVRs encoded genes that can affect human reproductive ability (such as genes from the gene clusters of *DAZ*, *CDY*, and *XKRY*) and may play an important role in high-altitude adaptation of Tibetans [1].

**Table 2:** CNVRs with Entrez gene and significant difference in allele frequency.

Chr	Start-Stop (kb)	Size (bp)	Type	Allele frequency				Modified *P* b	Entrez gene list
				Tibetan	EA^a^	CEU	YRI		
4	128908–129039	130875	deletion	10.34	NR	NR	NR	/	*SLC25A31,PLK4*
4	69047–69210	162544	deletion	75.86	32.85	55.56	38.89	3×10^−6^	*UGT2B17,UGT2B15*
5	68845–70959	2113387	deletion	13.79	NR	NR	NR	/	*OCLN,GTF2H2,SERF1A,SERF1B,SMN1,SMN2,NAIP,BDP1,MCCC2*
6	32561–32767	205223	deletion	6.90	67.18	92.22	98.89	3×10^−11^	*HLA-DRB5,HLA-DRB1,HLA-DQA1,HLA-DQB1*
7	101901–102109	208600	deletion	20.69	NR	NR	NR	/	*POLR2J,POLR2J2,POLR2J3,RASA4*
11	48346–49011	664649	duplication	41.38	2.17	5.56	0.00	2×10^−26^	*OR4A47*
15	18811–20093	1281179	multi-allele	27.59	71.36	71.11	52.22	1×10^−06^	*OR4M2,OR4N4,OR4N3P*
17	18855–19017	162199	deletion	31.03	NR	NR	NR	/	*GRAP*
22	21199–21591	391792	deletion	17.24	NR	NR	NR	/	*ZNF280A,PRAME,POM121L1P,GGTLC2*
X	61647–64531	2884335	duplication	44.83	4.09	55.56	58.89	2×10^−24^	*ARHGEF9,FAM123B,ZC4H2,ASB12,MIR1468,MTMR8*
X	47756–47870	113782	deletion	6.90	50.00	51.11	58.89	9×10^−05^	*ZNF630,SSX6*
Y	23611–24132	520913	duplication	20.69	66.67	53.33	43.33	4×10^−05^	*DAZ1,DAZ2,DAZ3,DAZ4*
Y	24259–27226	2967515	duplication	17.24	67.78	53.33	43.33	5×10^−06^	*TTTY3,TTTY4,TTTY17A,CDY1,CDY1B,BPY2,BPY2B,BPY2C,DAZ2,DAZ3,DAZ4*
Y	18179–19061	881529	duplication	34.48	NR	NR	NR	/	*XKRY,XKRY2,CDY2A,CDY2B*

Abbreviation: NR, not reported; kb, kilo base; bp, base pair; EA, East-Asian.

a the allele frequency was calculated with populations from East-Asian in Table 1 except Han Taiwanese [25] for data unavailable.

b *P* value was estimate from Tibetan and East Asian.

### Gene Ontology and Genetic Pathway Analyses

To further understand the functional implications of our CNVRs, in total, 184 Entrez genes from the 139 CNVRs were subjected to functional annotation and classification analysis with DAVID v6.7 [29]. We found 28 disease-related genes, classified into three classes: reproduction, cancer, and infection (Table 3). Cancer and infection related genes are frequently reported in other CNV studies [14], [30], [31], whereas human reproductive genes are rarely mentioned. Within these reproductive genes, *HLA-DRB1*, *HLA-DQA1*, and *HLA-DQB1* have been implicated in high infant birth weight and pregnancy loss [32], [33], and several genes present in the gene clusters of *DAZ*, *BPY2*, *CDY*, and *XKRY* have been reported to affect male germ cell development and fertilization [34], [35], [36]. The corresponding CNVRs of these reproductive genes exhibited lower frequencies in Tibetan than that in other EA populations (*P* values <0.009), as well as CEU (*P* values <0.0009) and YRI (*P* values <0.005).

**Table 3:** Genes in Tibetan CNVRs that are associated with disease risk.

CLASS	Genetic association diseases	Gene count	*P* value	Gene list
reproduction	infertility, male	10	0.0188	*BPY2B,HLA-DQB1,DAZ3,DAZ4,DAZ1,DAZ2,HLA-DRB1,BPY2C,BPY2,HLA-DQA1*
	pregnancy loss, recurrent			
	birth weight diabetes			
	type 1 head circumference at birth			
	birth weight body mass			
	birth weight gestational infections			
cancer	breast cancer	13	0.0238	*HLA-DQB1,MICA,HLA-DRB1,MRE11A,RRM2B,RAD52,HLA-DQA1,RAD51,UGT2B17,HLA-DRB5,SHC1,PGK1,UGT2B15*
	ovarian cancer			
	prostate cancer			
infection	HIV	9	0.0439	*HLA-DQB1,MICA,HLA-DRB1,CCL3L1,CCL3L3,CCL4L1,HLA-DRB5,CCL4L2,HLA-DQA1*
	arthritis, psoriatic			
	lupus erythematosus			
	rheumatoid arthritis			
	sclerosis, systemic			

By investigating enriched Gene Ontology categories, we found an overrepresentation of genes in categories of “sensory perception” (*P* values <0.0001) and “defense response to pathogens” (*P* values <0.008), which were also enriched in other CNV studies with East-Asian populations [22], [31]. Considering Tibetan-specific traits, we paid more attention to the categories of “female pregnancy,” “response to DNA damage stimulus,” and “DNA repair” (*P* values <0.004) (Table 4). The category of female pregnancy was enriched for the overrepresentation of the *PSG* gene family members. The allele frequencies of their corresponding CNVRs were similar between Tibetan and other EA populations. The last two categories contained 11 genes (*RAD51*, *RAD52*, *ANKRD17*, *MICA*, *EYA2*, *MRE11A*, *UBR5*, *RRM2B*, *ESCO2*, *GTF2H2*, and *ATRX*) corresponding to 10 CNVRs. It is worth noting that 7 of the 10 CNVRs were only observed in Tibetans.

**Table 4:** GO term enrichment of genes within Tibetan CNVRs.

GO term	GO category	Count	*P* Value	Genes
GO:0007600	sensory perception	20	1.12E-04	*OR4K5,OR4K2,OR4M2,OR4A47,LRTOMT,OR4C6,OR4M1, OR11H12,OR4K1,OR2T10,OR4S2,OR2T27,OR2T11,OR4C11, OR4N4,OR4Q3,OR2T35,OR4N2,OR4P4,ROM1*
GO:0042742	defense response to bacterium	11	5.22E-04	*DEFB107B,MICA,DEFB107A,DEFB106A,DEFB104B,SPAG11B, DEFB104A, DEFB103B,DEFB105B,DEFB105A,DEFB106B*
GO:0006974	response to DNA damage stimulus	11	0.002	*ATRX,ANKRD17,MICA,EYA2,MRE11A,UBR5,RRM2B, RAD52,ESCO2,GTF2H2,RAD51*
GO:0007565	female pregnancy	7	0.003	*PSG7, PSG6, PSG5, PSG4, PSG2, PSG1, PSG11*
GO:0006281	DNA repair	9	0.004	*ATRX,ANKRD17,EYA2,MRE11A,RRM2B,RAD52,ESCO2,GTF2H2, RAD51*
GO:0019882	antigen processing and presentation	5	0.007	*HLA-DQB1, MICA, HLA-DRB1, HLA-DRB5, HLA-DQA1*
GO:0009617	response to bacterium	11	0.008	*DEFB107B,MICA,DEFB107A,DEFB106A,DEFB104B,SPAG11B, DEFB104A,DEFB103B,DEFB105B,DEFB105A,DEFB106B*
GO:0033554	cellular response to stress	12	0.013	*ATRX,ATP7A,ANKRD17,MICA,EYA2,MRE11A,UBR5,RRM2B, RAD52,ESCO2,GTF2H2,RAD51*
GO:0007131	reciprocal meiotic recombination	3	0.020	*MRE11A,RAD52,RAD51*
GO:0006312	mitotic recombination	2	0.044	*RAD52,RAD51*

To further investigate the roles of CNVRs in high-altitude adaptation, we interrogated our data with annotations from the KEGG Pathway Database and Pathway Commons Database. Within all enriched pathways, we found 6 genes (*RAD51*, *RAD52*, *RRM2B*, *POLR2J*, *GTF2H2*, and *MRE11A*) involved in pathways related to DNA repair, such as “double-strand break repair,” “homologous recombination repair,” and “ATM-mediated response to DNA double-strand break”. Both results from genetic pathway and gene ontology analyses suggest that the DNA repair genes identified from our CNVRs may facilitate the adaptive trait of Tibetans.

## Discussion

Studies of Tibetan populations often provide knowledge of human migration and evolution for endurance of harsh natural conditions at high altitude. We generated a CNV profile with Tibetan genomes and added more understanding to the genetic diversity of Tibetans under the influence of high-altitude adaptation.

In this study, 139 putative CNVRs were found with a majority of them reported in DGV. Only 14 of them were newly identified CNVRs. We didn’t observe any Tibetan-specific common CNVRs (≥5%), which may provide clues to the migration and adaptation of Tibetan population. The detectable rate of a common CNVR was dependent on sample size. With only 29 Tibetan genomes, the Tibetan-specific common CNVRs may be under-ascertained here. Thus far, very few population-specific common CNVRs have been identified, even with large sample sizes [37]. A study using 3,578 Koreans reported that 15 out of 4,003 identified CNVRs were Korean-specific common CNVRs, with all frequencies less than 10% [22]. The lack of population-specific common CNVRs may be due to most *de novo* CNVRs being deleterious and having been eliminated by subsequent selection [11], [38].

For all the 139 CNVRs, the percentage of deletions overlapping with RefSeq genes is slightly higher than that of duplications (78% vs. 57%; *P*>0.05). Furthermore, a significant preference for overlapping of RefSeq genes in deletions over duplications (68% vs. 32%; *P*<0.02) was observed in a subset of the 139 CNVRs, which contained the 14 novel CNVRs and the 34 CNVRs showing allele frequency discrepancy with other EA populations. This preference is contrary to previous findings that deletions were found to contain less RefSeq genes than that of duplications [14], [39]. Some factors, such as under-ascertainment of small-size CNVs (<100 kb) with current microarrays, CNV reproducibility among different platforms and analytic tools [40], and different detection rates between duplication and deletion [14], [37], may influence our observations. However, our findings may be due to the need to improve fitness in high-altitude harsh environments. In this case, functional genes containing deletions may serve as substrates for natural selection in Tibetans [41], [42].

In the past two years, various beneficial genes, such as *EPAS1*, *EGLN1*, *ANGPT1*, and *PPARA*, were reported in studies of high-altitude adaptation [5], [9]. None of these genes were found to overlap with our CNVRs. Using all the identified Entrez genes (184) in this study, we conducted a functional annotation and classification analysis, and identified several enriched categories related to infant survival, such as birth weight, male infertility, and female pregnancy. One of the adaptive traits of Tibetans is high infant survival rate, which tightly correlates with heavier birthweight [1]. Genetic factors associated with heavier birthweight contribute to high-altitude adaptation. Our findings are consistent with previous results that the genes related to infant survival were under positive selection in Tibetans [5]. The enriched categories contain genes from the *DAZ*, *BYP*, and *HLA-DR* and *-DQ* gene clusters. Their corresponding CNVRs exhibited significant discrepancy in allele frequency between Tibetan and EA populations, which suggested important roles of these genes in recent adaptation of Tibetans.

The yield of radiation on Tibetan plateau is about two times higher than that found at sea level at the same latitude [43]. Darker skin tone is a distinctive trait of Tibetans and is mainly caused by high UV radiation. It is also known that UV radiation can lead to DNA damage [44] and impose a selection pressure on Tibetans. Within our CNVRs, at least 11 genes, such as *MRE11A*, *RAD51*, and *RAD52*, were involved in the gene ontology categories of “response to DNA damage stimulus” and “DNA repair” and genetic pathways of “double-strand break repair” and “homologous recombination repair”. All their corresponding CNVRs have low allele frequencies in Tibetans, but most of them are absent from other EA populations. In addition, three genes, *ATP7A*, *KGFLP2*, and *KGFLP1*, in epidermis morphogenesis, were also found in our CNVRs. These DNA repair and epidermis morphogenesis genes may be involved in UV radiation damage and play important roles in recent adaptation of Tibetans.

Our study presents a preliminary survey of copy number variation in Tibetans. We found that compared to other EA populations, Tibetans have a distinctive pattern of CNVR allele frequency distribution. The CNVRs are enriched for genes related to traits of infant survival rate and darker skin tone, thus, the suggestion of their involvement in local adaptation of Tibetans. Despite the need for more functional studies with larger sample size in the future, our results provide insights to high-altitude adaptation in Tibetans.

## Materials and Methods

### Ethics Statement

All donors signed informed consent forms for cell line establishment and subsequent biological investigations. This project was reviewed and approved by the Ethics Committee of the Beijing Institute of Genomics, Chinese Academy of Sciences.

### Samples and CNV Discovery

The peripheral blood samples of 29 unrelated Tibetans (16 males and 13 females) from Lhasa (at an altitude of 3700 meters) were used to prepare immortalized cell lines in this study. All DNA samples were obtained with DNA-extraction kits (Tiangen Biotech, Beijing, China) and genotyped on the Human 1M-Duo v3 chip (Illumina, CA, USA), according to the manufacturer's specifications. The microarray chip contained nearly 1.2 million SNPs and an additional 60,000 probes designed for CNV detection.

Genotyping modules from Genomestudio (Illumina, CA, USA) were employed to call the raw data. CNV Partition v2.3.4 (Illumina, CA, USA) was used to detect the presence of a CNV and to estimate the copy number of a CNV, based on log of the signal intensity (log R ratio) and B allele frequency measurement at each probe. Only CNVs detected with more than 5 probes with confidence scores greater than 35 were considered. Copy number variable regions (CNVRs) were defined after consolidation of all the overlapping CNVs. The CNVs obtained from this study were compared to the CNVs in the Database of Genomic Variants (http://projects.tcag.ca/variation/) and the CNVs from recent publications, using genotyping microarrays with Han Taiwanese, Han Chinese, and Koreans [20], [22], [23], [24], [25]. The *P* value of the allele frequency discrepancy was determined by a modified Chi-square test.

### Validation of CNVs

To validate CNVs identified by our chip method, we conducted a genomic real-time quantitative PCR with the 7500 Real-Time PCR system (Applied Biosystems, CA, USA). All novel CNVs were included for validation. The primers of the PCR are listed in supplementary material Table S2. To a 25-ul PCR reaction, 12.5 ul of SYBR Green Master mix (Applied Biosystems, CA, USA), 50 ng of genomic DNA, and 10 pmol of each primer were included. We validated our 16 novel CNVRs according to the method from D’Haene *et al* [45]. For each validation, the copy number of a CNVR was determined by the qPLUS software with the use of *ZNF80* and *GPR15* for normalization and a sample with copy number variation equal 2 as positive control and water as negative control. Each reaction was performed in replication. Of all 16 novel CNVRs, 14 of them were validated by quantitative PCR. We did not remove them from analysis for their neglectable effect on statistics.

### Gene Set Enrichment Analysis

We submitted all 184 Entrez genes that overlapped with our CNVRs to DAVID v6.7 for functional annotation and classification analysis [29]. The *P* value of gene set enrichment was calculated by a modified Fisher’s exact test. We kept GO terms and disease classes with *P* value less than 0.05. As for the pathway enrichment analysis, Web-based Gene Set Analysis Toolkit 2.0 was used with the pathway annotation from the KEGG Pathways Database and the Pathway Commons Database [46].

## Supporting Information

Table S1 **Details of CNVRs and CNVs observed in Tibetans.**
(XLS)

Table S2 **Genomic qPCR validation of the 16 novel CNVRs identified in Tibetans.**
(XLS)
